# Translational evidence for two distinct patterns of neuroaxonal injury in sepsis: a longitudinal, prospective translational study

**DOI:** 10.1186/s13054-017-1850-7

**Published:** 2017-10-23

**Authors:** Johannes Ehler, Lucinda K. Barrett, Valerie Taylor, Michael Groves, Francesco Scaravilli, Matthias Wittstock, Stephan Kolbaske, Annette Grossmann, Jörg Henschel, Martin Gloger, Tarek Sharshar, Fabrice Chretien, Francoise Gray, Gabriele Nöldge-Schomburg, Mervyn Singer, Martin Sauer, Axel Petzold

**Affiliations:** 1 Department of Anesthesiology and Intensive Care Medicine, University Medical Center Rostock, Rostock, Germany; 2 Bloomsbury Institute of Intensive Care Medicine, University College London, London, UK; 3 Department of Pathology, UCL Institute of Neurology, University College London, London, UK; 4 Department of Neurology, University Medical Center Rostock, Rostock, Germany; 5 Institute for Diagnostic and Interventional Radiology, University Medical Center Rostock, Rostock, Germany; 6 Department of Internal Medicine, Intensive Care Unit, University Medical Center Rostock, Rostock, Germany; 7 General Intensive Care Medicine, Assistance Publique – Hôpitaux de Paris, Raymond Poincaré Hospital, University of Versailles Saint-Quentin en Yvelines, Paris, France; 8 Department of Neuroimmunology, UCL Institute of Neurology, University College London, Queen Square, London, WC1N 3BG UK

**Keywords:** Intermediate filaments, Biomarkers, Animal models, Rats, Sepsis, Encephalopathy, Sepsis-associated encephalopathy, SAE

## Abstract

**Background:**

Brain homeostasis deteriorates in sepsis, giving rise to a mostly reversible sepsis-associated encephalopathy (SAE). Some survivors experience chronic cognitive dysfunction thought to be caused by permanent brain injury. In this study, we investigated neuroaxonal pathology in sepsis.

**Methods:**

We conducted a longitudinal, prospective translational study involving (1) experimental sepsis in an animal model; (2) postmortem studies of brain from patients with sepsis; and (3) a prospective, longitudinal human sepsis cohort study at university laboratory and intensive care units (ICUs). Thirteen ICU patients with septic shock, five ICU patients who died as a result of sepsis, fourteen fluid-resuscitated Wistar rats with fecal peritonitis, eleven sham-operated rats, and three human and four rat control subjects were included. Immunohistologic and protein biomarker analysis were performed on rat brain tissue at baseline and 24, 48, and 72 h after sepsis induction and in sham-treated rats. Immunohistochemistry was performed on human brain tissue from sepsis nonsurvivors and in control patients without sepsis. The clinical diagnostics of SAE comprised longitudinal clinical data collection and magnetic resonance imaging (MRI) and electroencephalographic assessments. Statistical analyses were performed using SAS software (version 9.4; SAS Institute, Inc., Cary, NC, USA). Because of non-Gaussian distribution, the nonparametric Wilcoxon test general linear models and the Spearman correlation coefficient were used.

**Results:**

In postmortem rat and human brain samples, neurofilament phosphoform, β-amyloid precursor protein, β-tubulin, and H&E stains distinguished scattered ischemic lesions from diffuse neuroaxonal injury in septic animals, which were absent in controls. These two patterns of neuroaxonal damage were consistently found in septic but not control human postmortem brains. In experimental sepsis, the time from sepsis onset correlated with tissue neurofilament levels (*R* = 0.53, *p* = 0.045) but not glial fibrillary acidic protein. Of 13 patients with sepsis who had clinical features of SAE, MRI detected diffuse axonal injury in 9 and ischemia in 3 patients.

**Conclusions:**

Ischemic and diffuse neuroaxonal injury to the brain in experimental sepsis, human postmortem brains, and in vivo MRI suggest these two distinct lesion types to be relevant. Future studies should be focused on body fluid biomarkers to detect and monitor brain injury in sepsis. The relationship of neurofilament levels with time from sepsis onset may be of prognostic value.

**Trial registration:**

ClinicalTrials.gov, NCT02442986. Registered on May 13, 2015.

**Electronic supplementary material:**

The online version of this article (doi:10.1186/s13054-017-1850-7) contains supplementary material, which is available to authorized users.

## Background

Sepsis still carries a high incidence and mortality rate [1, 2]. Furthermore, sepsis-associated encephalopathy (SAE) in survivors can result in long-term physical, cognitive, and psychological impairment with high socioeconomic relevance [2, 3]. The condition is thought to be underrecognized [4, 5]. Current understanding of pathophysiological mechanisms of SAE is limited; more insights into this important field are needed [6, 7]. Animal models can shed some light on the complex pathophysiological mechanisms of SAE [8–10]. Microglial activation, mitochondrial dysfunction, oxidative stress, neuroinflammation, and neuronal apoptosis are potential risk factors for SAE, resulting in axonal degeneration [11–13]. The accumulation of β-amyloid peptide (Aβ), a part of the β-amyloid precursor protein (βAPP), forms neurotoxic amyloid plaques in the septic brain [14, 15]. The detection of misfolded proteins in the septic brain was linked to long-term cognitive deficits in rats [14]. Histologic demonstration of axonal injury by βAPP staining is now widely used in animal models and human neuropathology [16–18]. Beside advances in histology, in vivo diagnostics can detect axonal injury in SAE [19, 20]. Imaging and biomarker studies have been used to detect brain injury and predict neurologic outcome [21–23]. Nevertheless, both the primary mechanisms underlying SAE and the temporal development of SAE over the course of sepsis remain elusive [24]. We thus conducted a translational study to compare neuropathologic findings derived from postmortem brain samples of septic rats and humans with in vivo clinical and imaging results from patients with sepsis who had SAE. A correlation between newly formed septic brain lesions detected by neuroimaging with neuropathologic findings may provide better understanding of the temporal relationship between sepsis and the onset of SAE.

## Methods

### Rat model of sepsis-associated encephalopathy

All experiments were performed according to local ethics committee (University College London) and Home Office (UK) guidelines under the 1986 Scientific Procedures Act. Adult male Wistar rats (approximate body weight 300 g, 12–14 weeks old) were used to generate a 3-day in vivo sepsis model of fecal peritonitis. Sepsis induction and experimental procedures were performed as described in detail before [25]. Four naive (noninstrumented) rats, eleven sham-operated, and fourteen septic rats were included. All rats were instrumented under brief anesthesia with tunneled carotid arterial (left side) and jugular venous (right side) catheters to monitor hemodynamics, sample blood, and infuse fluids. The catheters were mounted onto a swivel-tether system, allowing the rat, on recovery from anesthesia, to have free movement in its cage and ad libitum access food and water. Sepsis was induced 24 h later by an intraperitoneal injection of fecal slurry (0.63 mg/100 g body weight, prepared from bowel contents of rats from the same batch). From 2 h postinsult, fluid resuscitation using a 1:1 solution of colloid and 5% glucose was administered at a rate of 10 ml/h for the first 24 h and then halved on successive days to ensure normovolemia and normoglycemia. All septic animals showed clinical signs of sepsis by decreased movements, decreased alertness, hunched posture, and piloerection from about 12 h postinjection of fecal slurry. Rats were killed by cervical dislocation under deep isoflurane anesthesia either 24, 48, or 72 h after sepsis induction. After craniectomy, brains were removed within a few minutes of death. The brain was dissected through the midline into halves. One half was snap-frozen in liquid nitrogen; the other half was placed in formalin.

### Histology, immunohistochemistry, and protein extraction from septic rat and human brain

Neuropathologists (MG, FS) were masked to the condition of rat and human brain samples. Human and rat brain tissue from frontal lobe areas and the cerebellum were processed in paraffin wax using a standard 7-day dehydration and paraffin-embedding protocol on an automated tissue processor. Five-micrometer paraffin sections were cut, mounted onto Superfrost^TM^ glass slides and dried overnight at 37 °C. For the general histologic examination, sections were dewaxed, rehydrated, and stained with H&E using a standard protocol. For the immunohistochemical detection of neurofilaments (Nf) and glial fibrillary acidic protein (GFAP), the sections were dewaxed and rehydrated before being placed in 600 ml of 0.1 M citrate buffer (pH 6.0) and microwaved at full power for 15 minutes in a 850-W microwave oven. The four sections were allowed to cool before being rinsed in 0.05 M PBS, pH 7.4, and incubated overnight at room temperature in either a mouse monoclonal antibody directed against an epitope common to the 70 and 200 kDa Nf proteins (clone 2 F11 diluted 1:20; MP Biomedicals Inc., Santa Ana, CA, USA), β-tubulin (1:200; Sigma-Aldrich, Gillingham, UK), β-APP (1:500; Dako, Ely, UK), or a rabbit polyclonal antibody directed against GFAP (diluted 1:1500; Dako). After being washed in PBS, the sections were incubated in a biotinylated secondary antibody for 1 h (Dako), followed by a washing step and incubation in peroxidase-conjugated streptavidin for 1 h (diluted 1:300; Sigma-Aldrich) All dilutions were in PBS with 0.1% Triton-X. Antibody localization was visualized by incubating the sections for 10 minutes at room temperature in 0.05% diaminobenzidine with 0.04% NiCl_2_ and 0.01% hydrogen peroxide added. The sections were then counterstained with hematoxylin, dehydrated, cleared, and mounted.

The dry weight of the snap-frozen rat brain tissue was 0.56 to 1.94 g. Barbitone ethylenediaminetetraacetic acid (EDTA) buffer (pH 9.6) containing a protease inhibitor cocktail (P8340; Sigma-Aldrich) was added to 1:2 wt/vol. The samples were homogenized on ice using an ULTRA-TURRAX T 25 instrument (IKA-Werke GmbH & Co., Staufen, Germany) for 1 minute, followed by sonication on ice for 1 minute. Samples were refrozen at −70 °C and then thawed at 30 °C, and 2 ml of sample was added to 5 ml of diisopropyl ether and 2 ml of barbitone EDTA buffer. After a mixing step, the samples were spun at 25,000 × *g* and 4 °C for 30 minutes, and the protein soluble fraction was collected. Tissue levels of Nf heavy chain (NfH^SMI35^) and GFAP were measured by enzyme-linked immunosorbent assay (ELISA), and total protein was measured using the Lowry method [26, 27].

### In vivo neurologic assessment of patients with sepsis

The study was approved by the local ethics board at Rostock University (A 2012-0058) and registered as a clinical trial (ClinicalTrials.gov, NCT02442986). The patient recruitment period was from November 2012 to May 2015. All patients or their legal representatives signed written informed consent forms before study inclusion. Inclusion criteria for participants were aged ≥ 18 years and the presence of severe sepsis or septic shock according to the criteria used at the time [28]. Exclusion criteria were preexisting cerebrovascular diseases, including dementia, preexisting neuromuscular disease, high-dose glucocorticoid administration (>300 mg hydrocortisone or equivalent per day), preexisting renal replacement therapy, coagulopathy with active bleeding, and frequent administration of neuromuscular blocking agents (more than three times per week). Twenty patients with septic shock were included prospectively in the study. Seven participants without magnetic resonance imaging (MRI) examinations were excluded for the following reasons: death before MRI performed (*n* = 1), only cranial computed tomographic scan available owing to contraindication for MRI (*n* = 2), disclaimer for MRI from patient/legal representative after study inclusion (*n* = 2), and repeated surgery/unstable patient (*n* = 2). In total, 13 patients were enrolled prospectively in this single-center, longitudinal, observational study.

#### Clinical assessment protocol

All patients were clinically assessed by an interdisciplinary team consisting of intensivists and neurologists experienced in critical and neurocritical care using a validated scales for severity of disease: Acute Physiology and Chronic Health Evaluation II at ICU admission and the Sepsis-related Organ Failure Assessment score [29, 30]. All patients received standardized management according to guideline recommendations [2, 28]. After study inclusion, patients were longitudinally evaluated (study days 1, 3, 7, and 28) for their level of consciousness and for signs of SAE, such as confusion, agitation, hallucinations, or acute changes in mental status using the Glasgow Coma Scale, the Richmond Agitation-Sedation Scale, and the Confusion Assessment Method in the Intensive Care Unit (CAM-ICU) [31–34]. A medical history was taken from all patients, if obtainable, or from their next of kin. A standardized neurologic examination was performed on all patients by an experienced neurologist (MW). This comprised a detailed status of the level of consciousness; brainstem reflexes and function; deep tendon reflexes; and sensory and motor function, including muscular strength testing using the Medical Research Council dyspnea scale score [35, 36].

#### Electroencephalography

In addition to clinical assessment, all patients underwent electroencephalography (EEG) within the first 72 h after sepsis was diagnosed. The international 10–20 system was used for standard electrode placements with impedance level < 5 kΩ on a mobile EEG unit (ED 14; Madaus Schwarzer, Munich, Germany). All EEG recordings were done over 30 minutes. Patients were stimulated by verbal command. If no response to verbal stimulation could be obtained, sternal rub or nail bed compression were performed. The EEG recordings were assessed by an experienced accredited reader (MW) according to the method described by Young et al. [37]. Patients with analgosedation (standard regimen with continuous infusion of propofol and sufentanil) had a sedation holiday of 30 minutes before EEG recording.

#### Magnetic resonance imaging

A 1.5-T magnet system (MAGNETOM Avanto; Siemens Healthcare, Erlangen, Germany) was used in seven patients, and a 3.0-T magnet system (MAGNETOM Verio; Siemens Healthcare) was used in six patients. A standardized MRI protocol was used, and all MRI findings were analyzed by an experienced neuroradiologist (AG). MRI examinations included coronal T1-weighted images (with and without contrast medium), sagittal and axial T2-weighted sequences, axial fluid-attenuated inversion recovery (FLAIR), and axial T2*-weighted gradient recalled echo sequences. Additionally, axial echo planar imaging diffusion-weighted imaging (DWI) sequences, apparent diffusion coefficient maps, and time-of-flight magnetic resonance angiography were performed. The extent of white matter hyperintensities (WMH) as an imaging marker of brain injury was graded on a previously validated scale. WMH were scored according to their number and size from grade 0 (no lesions) through grade 1 (punctiform), grade 2 (patchy or confluent), and grade 3 (diffuse) [21, 38]. An MRI examination was performed as soon as the patient was clinically stable for in-house transfer using continuous patient monitoring (Expression MR400 monitor; Phillips Healthcare Deutschland GmbH, Hamburg, Germany).

### Statistical analysis

All statistical analyses were performed using SAS software (version 9.4; SAS Institute, Inc., Cary, NC, USA). Because of non-Gaussian distribution, the nonparametric Wilcoxon test was used for comparing two independent variables. Two-way unbalanced analysis of variance (general linear model) was used for comparing more than two independent variables, followed post hoc analysis if significance was achieved. The *F* values providing the degrees of freedom and the number of samples included in each particular analysis are shown. The linear correlation between continuous variables was evaluated using the Spearman correlation coefficient. Linear regression analysis was performed using the least squares method. A *p* value < 0.05 was considered significant.

## Results

### Experimental sepsis in rats

**Fig. 1:**
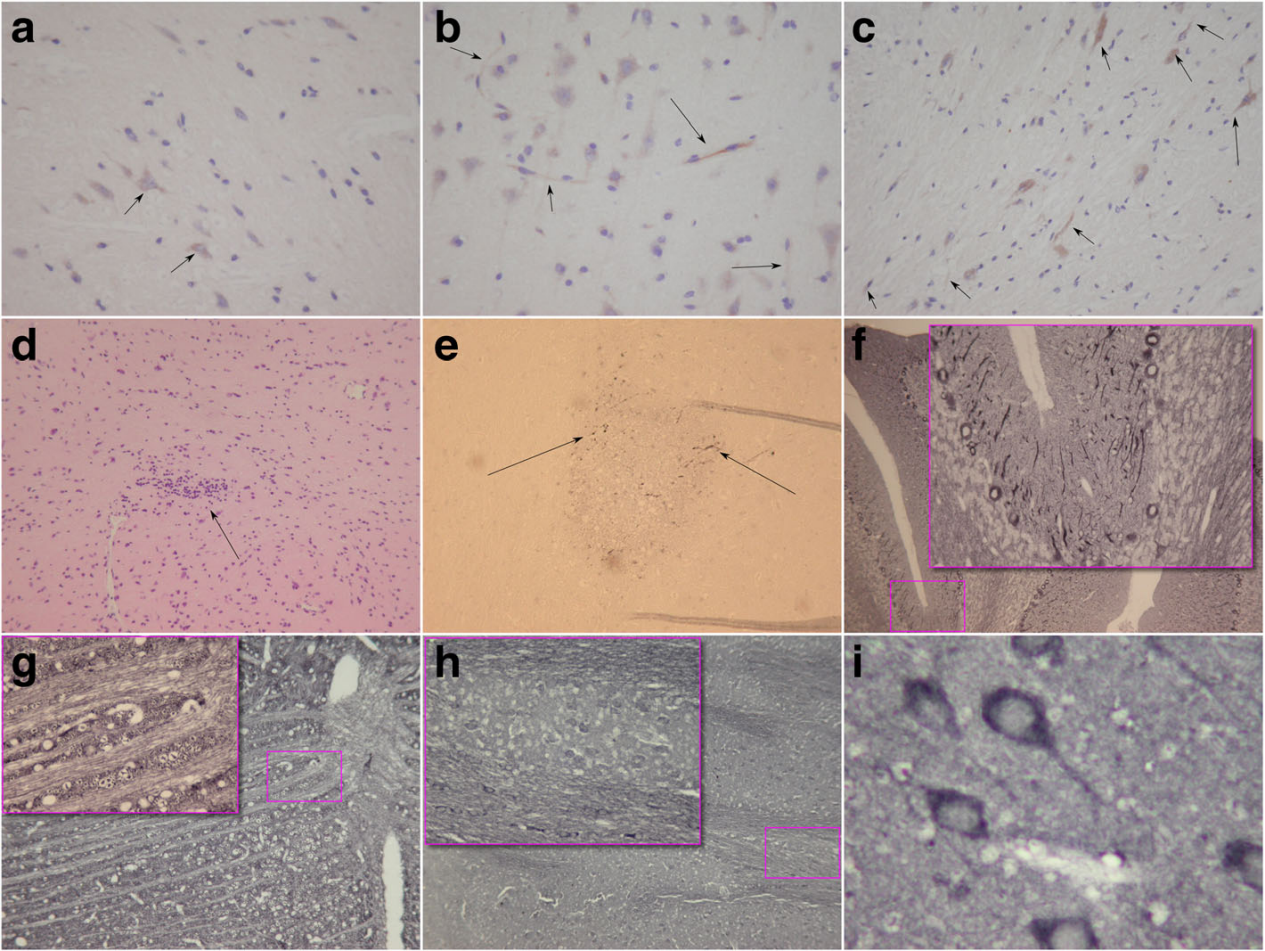
Brain lesions seen in rat sepsis model. **a** Central brain white matter immunohistochemistry in sham-treated animals (controls) shows characteristic neuronal soma with restricted staining for β-amyloid precursor protein (βAPP) (*arrows*). **b** In septic animals, brain tissue from the same locations showed abnormal and more widespread axonal staining extending from the axonal hillock to the proximal axon (*arrows*). **c** Abnormal axonal βAPP staining follows white matter tracts (*arrows*). **d** There are also pockets of inflammatory and ischemic brain lesions seen in the rat sepsis model (H&E stain; *arrow*). **e** Staining of such lesions shows intense neuronal and axonal staining for βAPP (*arrows*). **f** Staining of lesions of sham-treated animals for β-tubulin in the magnification field is crisp and shows integrity of the neuroaxonal compartment (overview, *inset*; original magnification × 10). **g** Likewise, the integrity of white matter tracts in sham-treated animals can be seen (×10; *inset*, original magnification × 40). **h** There is severe structural disorganization of the β-tubulin network in white matter tracts of the septic animals. **i** The level of structural β-tubulin disorganization in the septic rat brain is best observed at greater magnification (original magnification × 40) of the neuroaxonal compartment from the same location as that taken from the sham model shown in (**f**)

Average total protein levels were comparable between groups (naive 7.4 ± 2.6 g/L, sham 8.7 ± 3.9 g/L, and sepsis 8.9 ± 2.9 g/L). We found that brain tissue levels for GFAP were not statistically different when we compared sham-treated (0.27 ± 0.19 ng/g total protein) and septic (0.29 ± 0.21 ng/g total protein) rats with naive rats (0.34 ± 0.11 ng/g total protein). Average brain tissue levels of NfH^SMI35^ were higher in sham-treated (2.6 ± 2.2 ng/g total protein) and septic (1.8 ± 1.7 ng/g total protein) rats than in naive rats (0.8 ± 0.6 ng/g total protein), but this difference failed to reach statistical difference (*p* = 0.094 and *p* = 0.356, respectively) (Table 1). In septic rat brain tissue, there was a mild correlation between NfH^SMI35^ levels and time from sepsis induction (*R* = 0.53, *p* = 0.045) that was not seen for either GFAP (*R* = −0.39, *p* = 0.154) or total protein (*R* = −0.05, *p* = 0.854). No such correlations were observed in either sham-treated or naive rat brain samples. The immunohistochemistry of brain tissue from septic compared with naive and sham-treated rats showed two types of lesions. The normal white matter appearance in a sham-treated rat brain is shown in Fig. 1a. Typically, βAPP staining is restricted to the neuronal cell soma and the proximal axonal hillock. For comparison, in septic brain tissue, diffuse, more widespread axonal staining is seen (Fig. 1b). This pathologic staining was most marked for long white matter tracts (Fig. 1c). Spinal tissue was not available. The second lesion type seen comprised scattered small ischemic lesions (Fig. 1d). The early inflammatory component suggests a septic embolic etiology. These pockets of ischemic, inflammatory lesions stained intensely for βAPP, extending peripherally from the core lesion (Fig. 1e). An alternative marker for early axonal injury is β-tubulin. In sham-treated rats, the axonal staining for β-tubulin is neat and continuous, as expected on the basis of preserved neuronal (Fig. 1f) and axonal (Fig. 1g) integrity. This is best appreciated in the magnified insets in Fig. 1g and h. In contrast to the diffuse axonal pathology present in septic rat brains, axonal β-tubulin is subjected to proteolysis and broken up, giving the impression of a structurally disorganized axonal (Fig. 1h) and neuronal cytoskeleton (Fig. 1i).

**Table 1:** Brain tissue levels of total protein, glial fibrillary acidic protein, and neurofilament heavy chain in naive, sham-treated, and septic rats

Parameter	Naive group (*n* = 4)	Sham group (*n* = 11)	Septic group (*n* = 14)	*p* Value
Total protein levels, g/L	7.4 ± 2.6	8.7 ± 3.9	8.9 ± 2.9	>0.05
Brain tissue GFAP levels, ng/g total protein	0.34 ± 0.11	0.27 ± 0.19	0.29 ± 0.21	>0.05
Brain tissue NfH^SMI35^ levels, ng/g total protein	0.8 ± 0.6	2.6 ± 2.2	1.8 ± 1.7	>0.05

*Abbreviations: GFAP* Glial fibrillary acidic protein, *NfH* Neurofilament heavy chain, *Naive group* Controls (noninstrumented rats), *Sham group* Instrumented rats without injection of fecal slurry, *Septic group* Instrumented rats with injection of fecal slurry

### Human postmortem sepsis brain study

Postmortem brain tissue was available from five patients who died as a result of sepsis (mean age 64 years, one male patient). The three control subjects (mean age 37 years, all male) died as a result of other causes: suicide, assault, and road traffic accident (RTA) without concurrent brain injury.

**Fig. 2:**
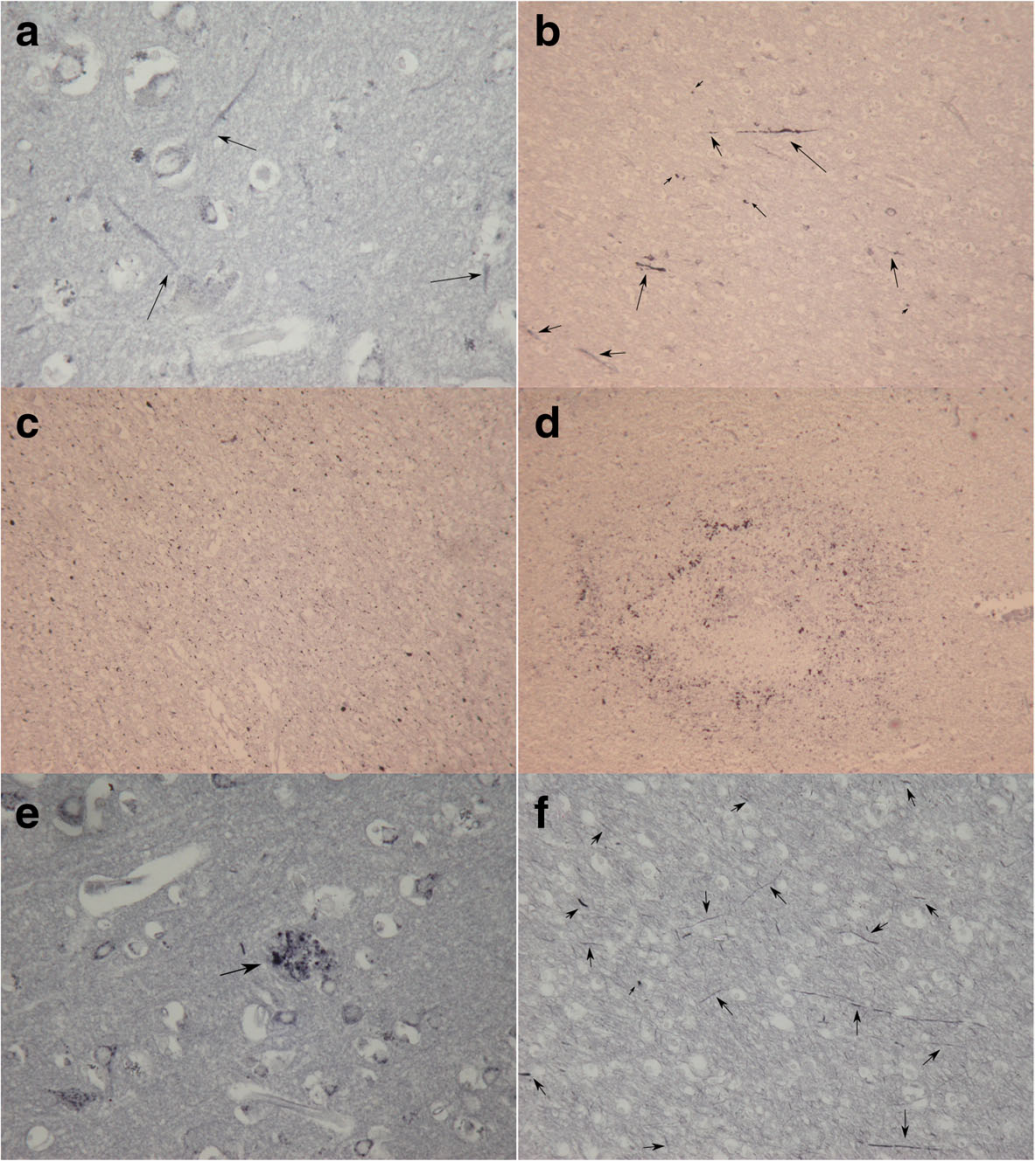
Brain lesions seen in postmortem human tissue. **a** Patient 1 (control): Diffuse staining of axons (*arrows*), extending from the axonal hillock (β-amyloid precursor protein [βAPP]). **b** Extensive diffuse axonal injury is shown in patient 2 (sepsis). Staining for βAPP is not restricted to the axonal hillock but is seen throughout the white matter tracts. Multiple axonal endbulbs can also be seen (*small arrowheads*). **c** Disruption of the deep white matter axons and the presence of axonal end bulbs are widespread based on dephosphorylated neurofilament heavy chain (SMI32). **d** Patient 3: Small areas of ischemic lesions can be seen throughout the brain (βAPP). **e** Patient 4: One type of lesion not observed in the animal model is shown. Amyloid plaques (arrow) are present and scattered throughout the brain tissue (βAPP). **f** In this patient, diffuse deep white matter axonal damage (arrows) is the most severe of this series (βAPP)

Causes leading to death in sepsis were directly related to multiorgan failure, but one patient had additional complications in the form of a gastrointestinal and intracranial hemorrhage. Careful examination of this patient’s brain did not reveal any evidence of an amyloid angiopathy. The immunohistochemical results of brain tissue from the patients with sepsis are summarized in Fig. 2. Patient 1, a 56-year-old male control subject, died after having an RTA. There was diffuse staining of axons for βAPP restricted to the axonal hillock (Fig. 2a, *arrows*). More extensive axonal βAPP staining was seen in patient 2, a 55-year-old male patient with sepsis (Fig. 2b). There was beadlike swelling indicating axonal pathology. In addition, staining for nonphosphorylated NfH (SMI32) demonstrated the presence of a large amount of axonal endbulbs, a sign of axonal degeneration (Fig. 2c). Next, scattered ischemic lesions were visible in patient 3, a 67-year-old female who died as a result of multiorgan failure due to sepsis (Fig. 2d). Amyloid plaques were found only in a 79-year-old female (Fig. 2e). Her medical history was not suggestive of cognitive impairment such as that seen with a neurodegenerative dementia; however, a formal neuropsychological assessment done before occurrence of sepsis was not available. Of note, the degree of diffuse deep white matter axonal injury was the most severe of all patients with sepsis (Fig. 2f).

### Clinical presentation of human septic brain injury in vivo

#### Clinical presentation of SAE and mortality in patients with sepsis

The baseline characteristics of the septic cohort are summarized in Table 2. In 10 of the 13 patients, clinical signs of SAE were present at the onset of sepsis. Three patients developed septic shock perioperatively, requiring continued ventilation and sedation and thus precluding reliable cognitive assessments. Longitudinal CAM-ICU scoring was positive in 8 of the 13 patients. Three patients died within the 100-day follow-up period.

**Table 2:** Clinical data of 13 patients with sepsis

Patient/sex	Age (years)	APACHE II/worst SOFA	Ventilation (days)	SAE at sepsis onset	Positive CAM-ICU during ICU stay	ICU stay (days)	Hospital stay (days)	Survival at day 100
1/F	63	20/18	72	Yes	Yes	72 (dead)	72 (dead)	No
2/F	82	29/15	12	Yes	Yes	22	30	Yes
3/M	73	42/15	20	n.a.	No	20	20	Yes
4/M	57	27/11	2	Yes	Yes	9	29	Yes
5/M	55	12/6	0	Yes	No	3	30	Yes
6/F	80	24/12	2	Yes	Yes	20 (dead)	20 (dead)	No
7/M	44	40/8	10	Yes	Yes	20	33	Yes
8/F	76	39/12	16	n.a.	Yes	16 (dead)	16 (dead)	No
9/F	74	23/13	9	Yes	No	21	31	Yes
10/M	75	37/10	2	Yes	Yes	4	26	Yes
11/F	54	48/11	8	n.a.	No	23	45	Yes
12/F	60	23/12	12	Yes	Yes	14	36	Yes
13/M	81	38/12	20	Yes	No	20	20	Yes

*Abbreviations: APACHE II* Acute Physiology And Chronic Health Evaluation II score at ICU admission, *CAM-ICU* Confusion Assessment Method in the Intensive Care Unit, *ICU* Intensive care unit, *n.a.* Not applicable (analgosedation), *SAE* Sepsis-associated encephalopathy, *SOFA* Sepsis-related Organ Failure Assessment

#### EEG findings in patients with sepsis

An EEG recording was done within a median of 2 days (range 0–4) after the onset of sepsis. At the time of recording, 7 of the 13 patients did not require sedation, and the remaining 6 had their sedation interrupted 30 minutes prior to undergoing EEG. EEG revealed encephalopathy of different extents in all patients (Table 3) Additional file 1.

**Table 3:** Results of magnetic resonance imaging and electroencephalography

Patient	Days from sepsis onset to MRI	MRI white matter hyperintensities	MRI ischemic lesions	Days to EEG	EEG abnormalities
1	27	Diffuse	No	4	Theta activity
2	8	Diffuse	Yes	1	Theta activity
3	7	None	No	4	Theta activity
4	7	None	No	3	Theta activity
5	9	Patchy/confluent	No	0	Theta activity
6	9	Punctiform	Yes	2	Delta activity
7	12	None	No	4	Delta activity
8	4	Punctiform	No	1	Delta activity
9	10	Patchy/confluent	No	2	Delta activity
10	10	Diffuse	No	2	Theta activity
11	17	None	No	4	Theta activity
12	4	Punctiform	No	1	Delta activity
13	5	Patchy/confluent	Yes	2	Delta activity

*EEG* Electroencephalography, *MRI* Magnetic resonance imaging

#### MRI findings in patients with sepsis

**Fig. 3:**
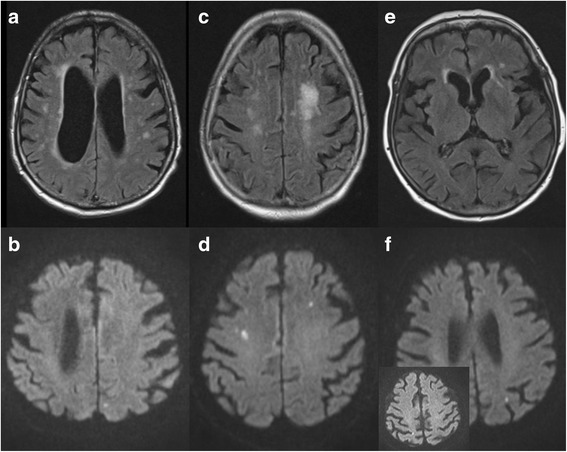
Brain magnetic resonance imaging of three patients during septic shock. The images are fluid-attenuated inversion recovery (FLAIR; **a**, **c**, and **e**) and echo planar imaging diffusion-weighted imaging (DWI; **b**, **d**, and **f**) scans. **a** and **b** An 81-year-old male patient with urosepsis. **a** Axial FLAIR image obtained on day 5 after the onset of septic shock shows punctiform and confluent white matter hyperintensities (WMH) in both paraventricular and paramedian regions (grade 2 leukoencephalopathy). **b** DWI study shows subacute ischemic lesion in the left occipital paramedian region. **c** and **d** An 80-year-old female patient with urosepsis. (**c**) Axial FLAIR image obtained 9 days after the onset of septic shock shows confluent WMH in the left periventricular region (grade 2 leukoencephalopathy). **d** DWI study shows bilateral ischemic lesions in the frontal region. **e** and **f** An 80-year-old female patient with urosepsis. **e** Axial FLAIR performed 8 days after onset of septic shock revealing a single punctiform WMH in the left periventricular region (grade 1 leukoencephalopathy). **f** DWI study shows punctiform ischemic lesions in the left occipital and parietal (*inset*) regions

Patients underwent MRI examination as soon as their clinical situation was deemed stable for in-house transfer. The median time from the onset of sepsis to brain MRI was 9 days (range 4–27). Reasons for delayed time to MRI were repeated surgical interventions in patients 1 and 11 and organizational reasons in patient 7. The MRI showed brain injury in 9 of 13 patients (Table 3, Fig. 3). The first pattern of WMH was punctiform (*n* = 3), patchy/confluent (*n* = 3), or diffuse (*n* = 3). The second pattern of WMH was ischemic (*n* = 3) (Table 3, Fig. 3).

## Discussion

The present translational study was performed to demonstrate evidence of axonal injury in the septic brains of animals and humans. Previous animal studies offer some evidence for different pathologic mechanisms of septic brain injury and behavioral changes [8–10, 12, 14, 39]. The histologic findings in the rat brains in the present study suggest two major mechanisms of injury. By βAPP staining, both diffuse axonal injury (DAI) and ischemic lesions were visible in the septic rat brain but not in sham-treated animals. Furthermore, βAPP staining showed abnormal axons along the white matter tracts. Pockets of inflammatory and ischemic lesions were detected by H&E staining. Further evidence for brain lesions in terms of disorientation of white matter was seen in β-tubulin staining of the septic animals but not the sham-treated group.

Our postmortem histologic samples of human septic brains showed results comparable to those derived from animal studies. Both βAPP staining and immunohistology for nonphosphorylated NfH monoclonal antibodies (SMI32) indicated DAI. Amyloid plaques were found only in human septic brains with βAPP staining, but they were completely absent in the rat histology. In contrast, Schwalm et al. reported increased Aβ levels in experimental sepsis [14]. Sharshar et al. noted various cerebral pathologies in patients who died as a result of sepsis, including ischemic lesions and neuronal apoptosis [40, 41]. The results of our present study are therefore in line with these previous reports, although our neuropathologic findings point to ischemia and DAI as the main mechanisms of brain injury. Owing to a longer interval from death to brain sampling in the patients, the histologic detection of DAI could represent a neuropathologic artifact. The importance of amyloid plaques within the human but not the animal brain histology is uncertain. These plaques could have predated the fatal sepsis episodes. Currently, on one hand, it can only be speculated that patients with amyloid plaques have a higher risk of developing SAE. Amyloid plaques, on the other hand, could be the result of a severe inflammatory stimulus to the brain, resulting in neurodegeneration and SAE, as shown before [14].

A limitation of both the human postmortem and experimental histologic studies was that investigations were restricted to brain tissue. There was no assessment of spinal cord tissue. The longest and, for DAI, most vulnerable axons travel through the spinal cord white matter tracts. We have previously demonstrated spinal cord involvement in a postmortem study of patients who died following West Nile virus infection [42]. Therefore, this study falls short of a conclusive demonstration of the anatomical distribution of diffuse white matter tract axonal injury in sepsis. Another limitation in this context is the lack of quantitative neuropathology. A potential future study may need to consider to correlate histological neuron/axon count with tissue body fluid levels [43].

It would clearly be useful to diagnose brain injury in patients with sepsis as early as possible for neurologic prognostication [21, 44]. Compared with improvements within neuropathology to detect DAI, routinely available in vivo diagnostics still lack the precision to accurately diagnose brain injury [45, 46]. In all clinically assessable patients with sepsis in the present study, clinical signs of SAE were present at the onset of sepsis, and this is consistent with pathologic EEG findings, as reported before [37, 47]. Nevertheless, the value of results derived from EEG examinations in the ICU setting is still under debate [19]. There was no statistically significant correlation between EEG and MRI results. We found that delirium screening systems such as CAM-ICU could not reliably detect SAE at all study points, owing to a high proportion of patients requiring sedation and mechanical ventilation. A clinical bedside diagnosis of SAE is not routinely feasible and is a well-recognized shortcoming of clinical assessment in ICU patients [19, 20, 48]. Specific biomarkers for neuroaxonal injury could be helpful in diagnosing DAI or SAE in vivo [49]. Previous clinical studies have been focused on neuron-specific enolase and S100B to diagnose brain injury in sepsis, with heterogeneous results [23]. Elevated Nf levels as markers of axonal injury were detected in various neurological conditions and may be of future use in SAE [49, 50].

In line with previous reports [21, 22], cerebral MRI did indicate brain injury in the majority of our patients. MRI findings suggested different extents of acute brain injury. In addition to WMH, acute or subacute ischemic lesions were detected in some of our patients. Compared with diffusion tensor imaging (DTI), conventional MRI techniques underestimate the extent of DAI [51, 52]. We can only hypothesize about more extensive axonal injury in our patients because they were examined only by MRI. Consistent with our postmortem neuropathological findings, the in vivo MRI results confirmed axonal and ischemic brain injury in our patients. We assume the inflammatory pockets in the postmortem histology to be correlates to the punctiform WMH seen on MRI scans. Although patients with any preexisting central nervous system pathology were not included in our study, we cannot exclude that some patients might have had subclinical brain injury before study inclusion that was interpreted as newly detected WMH. However, the ischemic lesions seen on MRI scans were undoubtedly newly present and provide striking evidence for ischemic brain injury during sepsis.

## Conclusions

Axonal and ischemic brain injuries were detected in septic rat and postmortem human brain neuropathology and appear to be important mechanisms underlying SAE. The MRI findings of ischemic lesions and WMH were the best correlates to neuropathology. For in vivo detection of axonal injury in SAE, Nf body fluid levels should be analyzed longitudinally. DTI could be an option for detecting DAI in vivo more appropriately and should be considered in further clinical studies.

## Additional file

Additional file 1: EEG delta activity. (PNG 640 kb)

## Abbreviations

Aβ: β-Amyloid peptide
APACHE II: Acute Physiology and Chronic Health Evaluation II
βAPP: β-Amyloid precursor protein
CAM-ICU: Confusion Assessment Method in the Intensive Care Unit
DAI: Diffuse axonal injury
DTI: Diffusion tensor imaging
DWI: Diffusion-weighted imaging
EDTA: Ethylenediaminetetraacetic acid
EEG: Electroencephalography
ELISA: Enzyme-linked immunosorbent assay
FLAIR: Fluid-attenuated inversion recovery
GFAP: Glial fibrillary acidic protein
MRI: Magnetic resonance imaging
Nf: Neurofilaments
NfH: Neurofilament heavy chain
RASS: Richmond Agitation-Sedation Scale
RTA: Road traffic accident
SAE: Sepsis-associated encephalopathy
SOFA: Sepsis-related Organ Failure Assessment
WMH: White matter hyperintensities
