# Supplementary figures and images for: Translational evidence for two distinct patterns of neuroaxonal injury in sepsis: a longitudinal, prospective translational study
Source: Crit Care. 2017 Oct 23;21:262. doi: 10.1186/s13054-017-1850-7 (PMC5653470; doi:10.1186/s13054-017-1850-7)

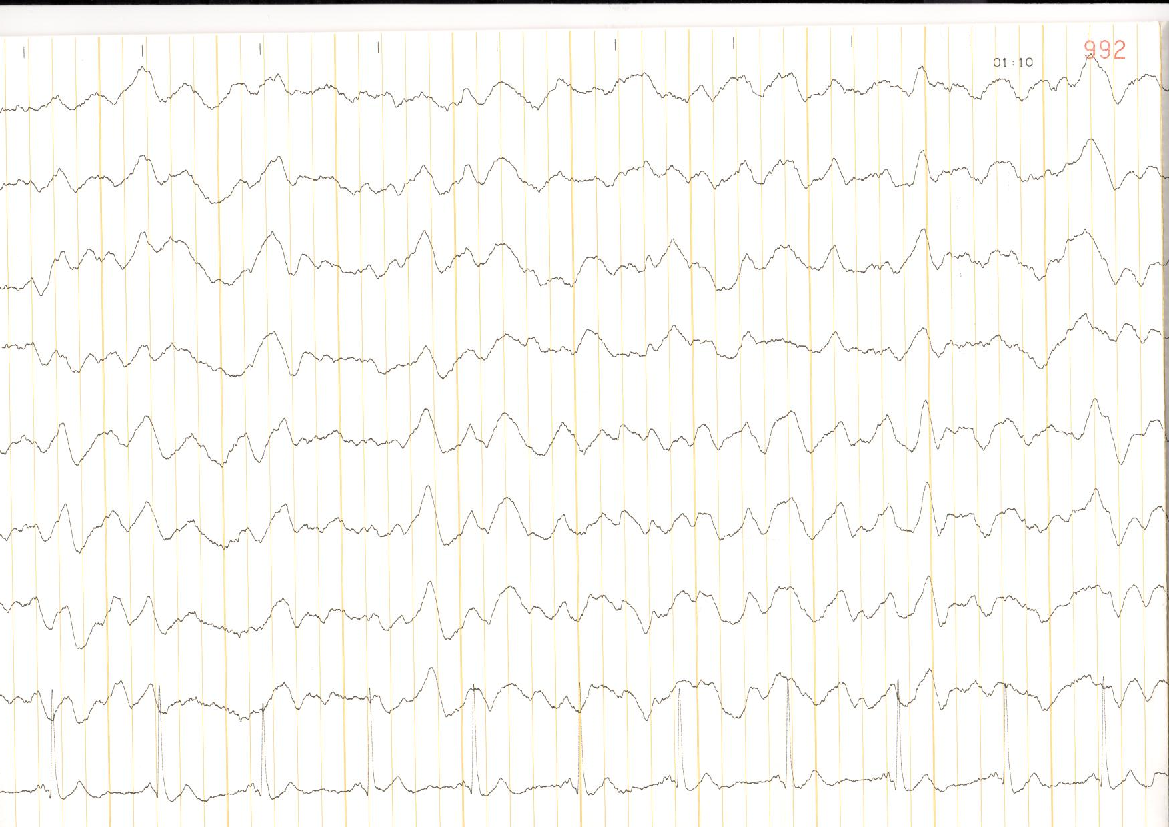

Supplement: Additional file 1: — EEG delta activity. (PNG 640 kb) [file 13054_2017_1850_MOESM1_ESM.png]
